# Comparison of Enzymatic Traits between Native and Recombinant Glycine Sarcosine *N*-Methyltransferase from *Methanohalophilus portucalensis* FDF1^T^

**DOI:** 10.1371/journal.pone.0168666

**Published:** 2016-12-30

**Authors:** Shu-Jung Lai, Yu-Chen Deng, Mei-Chin Lai

**Affiliations:** 1 Department of Life Sciences, National Chung Hsing University, Taichung, Taiwan; 2 Institute of Biological Chemistry, Academia Sinica. Taipei, Taiwan; 3 Agricultural Biotechnology Center, National Chung Hsing University, Taichung, Taiwan; Leibniz-Institut fur Pflanzengenetik und Kulturpflanzenforschung Gatersleben, GERMANY

## Abstract

The halophilic methanoarchaeon *Methanohalophilus portucalensis* FDF1^T^ possesses the ability to synthesize the osmolyte betaine from its precursor, glycine, in response to extracellular salt stress through a three-step transmethylation process. Analysis of recombinant glycine sarcosine *N*-methyltransferase (rGSMT) and recombinant sarcosine dimethylglycine *N*-methyltransferase (rSDMT) from *Escherichia coli* indicated that betaine synthesis is rate-limited by rGSMT and is constitutively activated by rSDMT. Therefore, it is of interest to purify native GSMT from *Methanohalophilus portucalensis* to further compare its enzymatic characteristics and kinetics with rGSMT. In this study, native GSMT was purified through DEAE ion exchange and gel filtration chromatography with 95% purity. The enzymatic characteristics of GSMT and rGSMT showed similar trends of activities that were activated by high concentrations of monovalent cations. Both were feedback-inhibited by the end product, betaine, and competitively inhibited by *S*-adenosylhomocysteine (SAH). Native GSMT was 2-fold more sensitive to SAH than rGSMT. Notably, comparison of the kinetic parameters illustrated that the turnover rate of glycine methylation of GSMT was promoted by potassium ions, whereas rGSMT was activated by increasing protein-glycine binding affinity. These results suggest that GSMT and rGSMT may have different levels of post-translational modifications. Our preliminary mass spectrometry evidence indicated that there was no detectable phosphosite on GSMT after the complicated purification processes, whereas purified rGSMT still possessed 23.1% of its initial phosphorylation level. We believe that a phosphorylation-mediated modification may be involved in the regulation of this energy consuming betaine synthesis pathway during the stress response in halophilic methanoarchaea.

## Introduction

The halophilic methanoarchaeon *Methanohalophilus portucalensis* FDF1^T^ is a model strain for investigating the strategy of acclimation under salt and temperature stresses because it was isolated from a solar saltern, where it has a wide range of temperature gradients and salt concentrations [1]. One strategy used to adapt to salt and osmotic stresses is the accumulation of potassium ions and small molecular osmolytes (compatible solutes), including β-amino acid derivatives, β-glutamate, β-glutamine, and N^ε^-acetyl-β-lysine, which are observed ubiquitously in methanoarchaea [2–4]. The osmolyte glycine betaine (betaine) possesses the highest osmoprotective efficiency and can be taken up as a compatible solute to encounter salt, osmotic and cold stresses among three organismal domains [3, 5–7]. Betaine is actively transported via BtaABC (betaine transporter in archaea) [8], and two open reading frames of a sodium/proton forced choline/carnitine/betaine transporter were identified in the *M*. *portucalensis* FDF1^T^ genome (Lai et al., unpublished data). Additionally, betaine can be synthesized from its precursor, glycine, via a three-step methylation process with S-adenosylmethionine (SAM), a methyl donor, by glycine sarcosine *N*-methyltransferase (GSMT, AEG64703) and sarcosine dimethylglycine *N*-methyltransferase (SDMT, AEG64704) to produce the intermediates sarcosine and dimethylglycine; the end product, betaine; and a competitive inhibitor, S-adenosylhomocysteine (SAH) [8–13]. Although many halophilic or halotolerant bacteria can synthesize betaine by a two-step oxidation pathway, only extreme halophilic bacteria, including *Actinopolyspora halophila*, *Halorhodospira halochloris*, *Aphanothece halophytica*, non-halophilic *Synechococcus* sp. WH8102, and *Myxococcus xanthus*, possess GSMT and SDMT/DMT, which are required for betaine synthesis [14–18].

Our previous studies showed that *M*. *portucalensis* FDF1^T^ possesses two betaine synthesis enzyme systems, GSDMT [10] and GSMT/SDMT [9, 11], which are unique among all known isozymes from halophilic/halotolerant bacteria. It is noteworthy that GSDMT from *M*. *portucalensis* is the only amine *N*-methyltransferase with broad specificity for the substrates glycine, sarcosine, and dimethylglycine [10], whereas the other system exhibits partially overlapping substrate specificity between GSMT and SDMT/DMT [14–18]. The activity of recombinant MpGSMT (rGSMT) is activated by increasing monovalent ions to adapt to osmotic stress and is feedback-inhibited by the end product betaine to conserve energy [11]. Recombinant MpSDMT (rSDMT) is a constitutive enzyme used to produce the osmolyte betaine, which is limited by the amount of the substrate, sarcosine, and the inhibitor, SAH [11].

Recently, phosphoproteomic analysis of *M*. *portucalensis* FDF1^T^ showed that both GSMT and SDMT are post-translationally modified by phosphorylation [19]. According to our previous data, native SDMT showed lower substrate binding affinity than rSDMT, whereas the reaction velocity of SDMT was higher than that of rSDMT [9, 11]. We hypothesize that this phenomenon may be due to the substrate specificity of protein kinases from *M*. *portucalensis* FDF1^T^ and the recombinant host *Escherichia coli*, which causes different levels of phosphorylation between SDMT and rSDMT. In this study, we purified native GSMT from *M*. *portucalensis* FDF1^T^ for enzymatic comparison with rGSMT. The phosphorylation sites of GSMT and purified rGSMT were identified through liquid chromatography-tandem mass spectrometry (LC-MS/MS) to elucidate the different modifications between native GSMT and rGSMT.

## Materials and Methods

### Organism and growth conditions

The halophilic methanoarchaeon *M*. *portucalensis* FDF1^T^ (DSM 7471) was routinely cultured in H-P-defined medium containing 2.1 M NaCl and 20 mM trimethylamine as the carbon and energy source, respectively [1, 3]. The medium was prepared under a N_2_:CO_2_ atmosphere (4:1) to eliminate oxygen. *Methanohalophilus portucalensis* FDF1^T^ cultures were inoculated at a 1:20 (v/v) ratio in fresh anaerobic H-P medium containing 0.025% Na_2_S, which was added to the medium as a reductant under a modified Hungate technique [20]. Cultures were incubated at 37°C until late log phase (OD_540_ value 0.7~0.8) and then aerobically harvested by centrifugation (Sorvall RC-5C, DuPont, Wilmington, DE, USA) at 12,100 x g for 15 min at 4°C. The cell pellet from 4.8 L of *M*. *portucalensis* FDF1^T^ cultures was stored at -80°C.

### Cell extract preparation

The cell pellet of *M*. *portucalensis* FDF1^T^ was resuspended in buffer A (50 mM Tris-base, 1 mM EDTA, 1 mM β-mercaptoethanol, pH 7.3) with the addition of 1 mM phenylmethylsulfonyl fluoride (PMSF) as a protease inhibitor and was incubated on ice for 30 min. The suspended *M*. *portucalensis* FDF1^T^ cells were disrupted using an ice-cold French press (French pressure Cell Press, SLM Instruments, Inc.) with 10,000 psi four to five times. The cell extract was harvested after centrifugation (Sigma 2k15, rotor 12139) at 12,557 x g for 30 min at 4°C to precipitate cell debris.

### Antigen rGSMT preparation for antibody production

To detect the native GSMT during each step of the purification process, rGSMT was expressed and purified for anti-rGSMT antibody preparation. Briefly, rGSMT was induced by the addition of 1 mM IPTG (final concentration) to the log phase (OD_600_ = 0.7) culture for 4 h. Cells were harvested by centrifugation (Sorvall RC-5C, DuPont Co.) at 12,100 x g for 15 min at 4°C and suspended in buffer A containing 1 mg/mL lysozyme and 1 mM PMSF for further disruption by a sonicator (Ultrasonic VXC750 sonicator) with a 6 sec burst at 40 W and 10 sec of cooling for a total of 10 min. Cellular debris was separated by centrifugation (Sigma 2k15, rotor 12139, 10,000 x g, 4°C) and the crude extracts (CEs) were harvested. rGSMT was purified using a one-step Ni Sepharose™ 6 Fast Flow column (Amersham Co.), which has been described previously (Lai and Lai, 2011). The above purified rGSMT (2 mg) was further injected into rabbits (*Oryctolagus cuniculus*) as an antigen four times by Quantum Biotechnology, Inc. (Taichung, Taiwan) to obtain the polyclonal anti-rGSMT antibody.

### Purification of native GSMT

All purification processes were performed at 4°C using ÄKTA^TM^ prime plus Fast Protein Liquid Chromatography (FPLC, GE Healthcare Bio-Sciences AB, Uppsala, Sweden). Based on the estimated pI value of GSMT (4.57), which was negatively charged in buffer A at pH 7.3, a HiPrep DEAE FF 16/10 ion exchange column (GE Healthcare Bio-Sciences AB) was chosen as the first step of the purification strategy. Approximately 170 mg of *M*. *portucalensis* FDF1^T^ crude extract was loaded onto a DEAE column immediately after equilibration using a 2-fold column volume (CV) of buffer A. The purification process began by washing out unbound proteins with a 2-fold CV of buffer A, followed by a linear gradient of buffer A that contained 0 to 0.25 M KCl for washing out unbound proteins. GSMT was eluted with a 10-fold CV of buffer A containing a linear gradient of 0.25 M to 0.45 M KCl, followed by washing out all other proteins with a 2-fold CV linear gradient of 0.45 M to 1.00 M KCl buffer A at a flow rate of 0.5 mL/min in each step. Fractions containing GSMT from four independent separations by DEAE chromatography were pooled. The pool of GSMT (29 KDa) was further enriched using Amicon^®^ Ultra-15 50 KDa Centrifugal Filter Devices (Millipore, Bellirica, MA, USA) to collect the flow-through fractions and then concentrated using Amicon^®^ Ultra-4 10 KDa Centrifugal Filter Devices (Millipore) at 4°C. In total, 2.25 mg/0.8 mL of GSMT-containing fractions was loaded onto a HiPrep 16/10 Sephacryl S-200 HR (GE Healthcare Bio-Sciences AB) gel filtration column following equilibration with a 1.5-fold CV (120 mL) of buffer A, and GSMT was eluted out at a fraction volume of 55~70 mL at a flow rate of 0.2 mL/min. The protein concentration was determined via standard Bradford protein assay processes (Bio-Rad) for estimating the concentration of purified GSMT.

### Gel electrophoresis

All purification fractions were analyzed by 12.5% SDS-PAGE with a PageRuler^TM^ Prestained Protein Marker (Fermentas Canada Inc., Ontario, Canada). The gel was stained with Coomassie Brilliant Blue (0.2% Coomassie Brilliant Blue R-250, 50% methanol, 7% glacial acetic acid) to estimate GSMT purity using TINA 2.09 software (Raytest, Straubenhardt, Germany). To strictly confirm the purity of GSMT, the proteins that were separated by 12.5% SDS-PAGE were visualized by silver staining. Briefly, proteins in the gel were fixed overnight with fixation solution (10% acetic acid, 30% ethanol) and incubated with gentle shaking in sensitized solution (30% ethanol, 0.2% w/v sodium thiosulfate [Na_2_S_2_O_3_], 17% w/v sodium acetate [CH_3_COONa], 0.125% w/v glutaraldehyde) for 30 min. The gel was rinsed with deionized water for 5 min, and this step was repeated three times. The gel was treated with silver reaction solution (0.25% w/v silver nitrate [AgNO_3_], 0.015% w/v formaldehyde) with gentle shaking for 20 min and then rinsed with deionized water for 1 min. Proteins were visualized immediately after treatment with developing solution (2.5% w/v sodium carbonate, 0.03% w/v formaldehyde) within 3 min, and the reaction was terminated by the addition of glacial acetic acid for 10 min.

### GSMT activity assays

Native GSMT methyltransferase activity assays were carried out using the acid-washed charcoal method following the standard procedures described previously [11]. According to the traits of rGSMT, whose activity is significantly improved by potassium ions [11], all GSMT activity assays, except for those indicated, were performed using a 0.1 M TES buffer system (pH 7.3) containing 1.0 M KCl. The reaction mixture (final volume 100 μL) consisted of 1 mM SAM (containing 0.1 μCi/2 pmol [methyl ^3^H]-SAM (PerkinElmer, Waltham, MA02451, USA)) as the methyl donor, which the radiolabeled methyl group transfers to the substrate glycine (0.5 M) or sarcosine (0.5 M). After incubation at 37°C for 1 h with 1 μg GSMT, the reaction was terminated by incubation with 25 μL of 10% trichloroacetic acid (TCA), whereas the radiolabeled sarcosine or dimethylglycine was separated from [methyl ^3^H]-SAM via precipitation with 125 μL of acid-washed charcoal (76 mg charcoal/1 mL 0.1 N acetic acid) in an ice-bath for 15 min and then centrifuged at 18,700 x g for 15 min at 4°C. The radioactivity of the supernatant (100 μL) was determined on a scintillant spectrometer (Pharmacia Co.) with 3 mL of counting scintillant (Ultima Gold^TM^, PerkinElmer). All data points shown in this study represent the average of triplicate experiments after subtracting the reaction without protein as a negative control.

### In-gel tryptic digestion and peptide recovery

To remove small molecules or compounds that may contaminate the protein samples for MS analysis, a total of 0.5 mg of purified GSMT or rGSMT was resolved via 12.5% SDS-PAGE (1.5-mm thick) and stained with InstantBlue™ Protein Stain (Expedeon, C.B.S. Scientific Co., Inc., USA). The GSMT and rGSMT bands were excised and further diced into small pieces approximately 1.0 mm^3^ in size to increase the surface area of each particle. All gel pieces were completely destained with 50% acetonitrile (ACN) in 25 mM ammonium bicarbonate, pH 8.5. ACN was then removed, followed by re-equilibration in 25 mM ammonium bicarbonate. The samples were treated with 30 mM DTT at 37°C for 1 h to reduce the protein and then treated with 60 mM iodoacetamide (IAA) at room temperature in the dark for 1 h for alkylation. The gel particles were dehydrated with 100% ACN and then freeze-dried. TPCK trypsin (1:50 w/w) (Pierce, Rockford, USA) was added to recuperate the original size and incubated at 37°C for 16 h, and the reaction was terminated by addition of 5% TFA/50% ACN. The resulting peptides were recovered twice with extraction buffer following incubation for 10 min in a sonication bath (Branson 1510, Branson Ultrasonic Corporation, Danbury, CT). Extracts were dried in a vacuum centrifuge and stored at −20°C.

### Nano LC-MS/MS analysis

Peptide mixtures were analyzed by online nanoflow liquid chromatography tandem mass spectrometry (LC-MS/MS) on a nanoAcquity system (Waters, Milford, MA) coupled to an LTQ-Orbitrap Velos hybrid mass spectrometer (Thermo Scientific) equipped with a PicoView nanospray interface (New Objective). Peptide mixtures were loaded onto a 75-μm × 250-mm nanoACQUITY UPLC BEH130 column packed with C18 resin (Waters, Milford USA) and separated at a flow rate of 300 nL/min using a linear gradient of 5 to 30% solvent B (95% acetonitrile with 0.1% formic acid) for 75 min, followed by a sharp increase to 90% B for 2 min, and held at 95% B for another 11 min. Solvent A was 0.1% formic acid in water. The effluent from the HPLC column was directly electrosprayed into the mass spectrometer. The LTQ Orbitrap Velos instrument was operated in data-dependent mode to automatically switch between full-scan MS and MS/MS acquisition. Instrument control was through Tune 2.6.0 and Xcalibur 2.1. Detailed settings for the Orbitrap analyzer followed a previous description [21].

### MS/MS database searching and phosphorylation site analysis

All MS and MS/MS raw data were analyzed using MaxQuant software (version 1.4.1.2) (http://www.maxquant.org/) with the built-in search engine Andromeda for phosphopeptide identification and phosphorylation site analysis [22–23]. The search criteria used for phosphopeptide and phosphosite analysis were as follows: trypsin digestion; cysteine carboxyamidomethylation (+57.0214 Da) as the fixed modification; methionine oxidation (+15.9949 Da); phosphorylation of serines, threonines, tyrosines, histidines, and aspartates; protein N-terminal acetylation as variable modification; up to two missed cleavages allowed; minimum of seven amino acids per peptide; and a mass accuracy of 10 ppm for the parent ion and 0.6 Da for the fragment ions. False discovery rates (FDRs), estimated from the target-decoy strategy, were used to distinguish correct and incorrect identifications. For identification, the FDR was set to 0.01 for both peptides and proteins.

## Results and Discussion

### Native GSMT purification process

**Fig 1:**
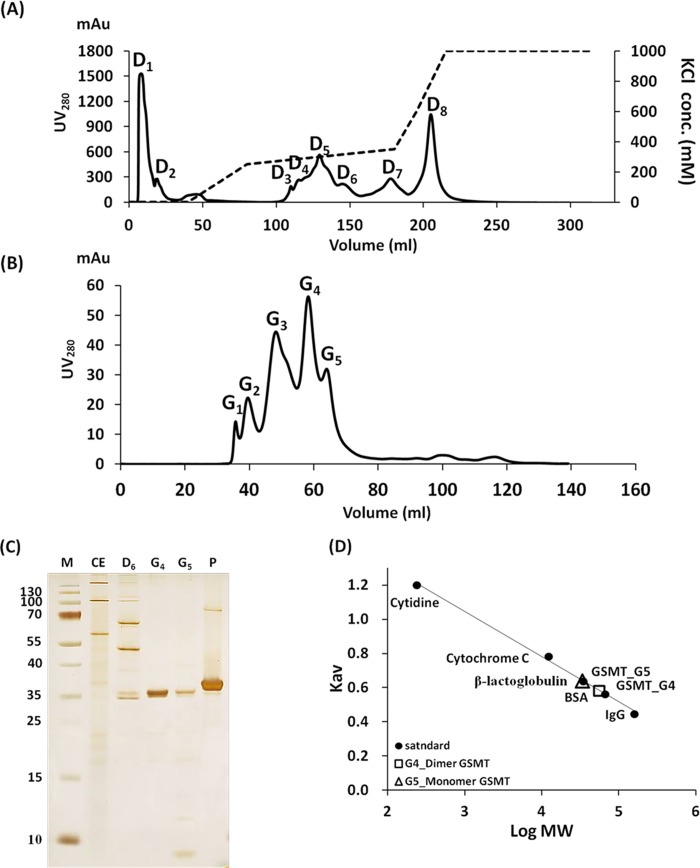
Native GSMT was purified by employing ion exchange and size exclusion chromatography. (A) FPLC profiles of Hiprep DEAE FF 16/10 Sepharose chromatography with step and linear gradients of KCl concentrations. The solid line indicates the absorbance at 280 nm, whereas the dashed line displays the KCl concentration. D1 to D8 indicate the peak numbers. (B) FPLC profiles of Hiprep 16/10 Sephacryl S-200 HR gel filtration chromatography. G1 to G5 indicate the peak numbers. (C) GSMT-containing protein fractions were separated by 12.5% SDS-PAGE, followed by silver staining. (D) Estimation of GSMT molecular weight via gel filtration with low-molecular-weight standard proteins, including IgG (160 KDa), BSA (67 KDa), β-lactoglobulin (35 KDa), cytochrome C (12.4 KDa), and cytidine (0.24 KDa).

From 4.8 L of *M*. *portucalensis* FDF1^T^, approximately 684.4 mg of protein was obtained from each batch. The enzyme GSMT was partially isolated through ion exchange chromatography with step and linear gradients of KCl concentrations (Fig 1A). Eight major peaks were identified following DEAE chromatography using the FPLC system (Fig 1A). GSMT eluted at peak D6 with 300–350 mM KCl was separated by 12.5% SDS-PAGE with silver nitrate stained (Fig 1C), and confirmed via Western blot analysis with an anti-rGSMT antibody. All protein fractions of D6 from four independent batches following FPLC-DEAE purification were pooled, and the monomeric GSMT was enriched by employing 50 K MW and then 10 K MW Amicon Centrifugal Filters. A total of 2.2 mg of the GSMT-containing protein fraction was loaded onto a Hiprep 16/10 Sephacryl S-200 HR gel filtration column using the FPLC system as the second purification step. Five major peaks were observed; GSMT was distributed in both peaks G4 and G5 (Fig 1B and 1C), which was confirmed by Western blot analysis with an anti-rGSMT antibody. The molecular weight of GSMT from peaks G4 (56 KDa) and G5 (34 KDa) was estimated by employing low-molecular-weight standard proteins, which suggested that they may represent the dimer (G4) and monomer (G5) forms, respectively (Fig 1D). Protein fractions of both G4 and G5 were pooled for concentrating using a 10 K MW centrifugal filter. The yield of GSMT from each purification step is listed in Table 1. Approximately 0.4 mg of native GSMT was obtained from each batch of the purification process with 95% purity, which was calculated by TINA software from signals on the 12.5% SDS-PAGE gel stained with Coomassie Blue.

**Table 1:** The yields and activities of GSMT purified from *Methanohalophilus portucalensis* FDF1^T^.

Purification process		Crude extract	DEAE Sepharose	10 MW Centrifugal Filter	Gel Filtration Chromatography
Total protein^a^ (μg)		684573	42128	2247	416
Specific activity^b^ (pmol / μg/ hr)	GMT	4.13±0.13	23.60±2.26	93.73±1.10	164.27±1.19
	SMT	4.49±0.24	22.58±3.09	82.72±0.18	129.46±0.76
Total activity^c^ (nmol/ hr)	GMT	2827.27	994.22	210.61	68.34
	SMT	3073.73	951.25	185.87	53.86
Purification fold^d^ (X-fold)	GMT	1.00	5.71	22.69	39.77
	SMT	1.00	5.03	18.42	28.83
Recovery^e^ (%)	GMT	100.00	35.17	7.45	2.42
	SMT	100.00	30.95	6.05	1.75
Purity^f^ (%)		3.00	35.00	53.00	95.00

a. A typical purification from 4.8 liter of *M*. *portucalensis* FDF1^T^ cultures. Total protein yields were estimated through the protein concentration multiplied by the amounts of volume obtained from each purification step. The standard deviation of protein concentration determination was less than 13%.

b. Enzyme activity was defined as pmol of transferred ^3^H-methyl group per μg of enzyme in 1 hour.

c. Total activity was estimated via specific activity multiplied by total protein obtained from each purification step.

d. Purification fold was measured by specific activity from each purification step versus the specific activity from crude extract.

e. Recovery was generated by total activity from each purification step versus the total activity from crude extract.

f. Purity % was determined using TINA software informer.

### Native GSMT was activated by monovalent cations

A previous study showed that the accumulation or efflux of potassium ions was the primary response of *M*. *portucalnesis* FDF1^T^ to immediately overcome the fluctuation in osmolarity; osmolytes then accumulated intracellularly via active transport or biosynthesis to balance the hyperosmotic pressure [3, 24]. The activities of rGSMT were enhanced at high concentrations of potassium or sodium ions [11], which is consistent with the traits of haloenzymes. This result also implied that the betaine biosynthesis mechanism was activated by intracellular potassium ions at the post-translational level. This characteristic was unprecedented in other published GSMT samples, even from halophilic *Ectothiorhodospira halochloris* and *Aphanothece halophytica*, whose activities were inhibited under the high salt (NaCl or KCl) condition [17–18].

**Fig 2:**
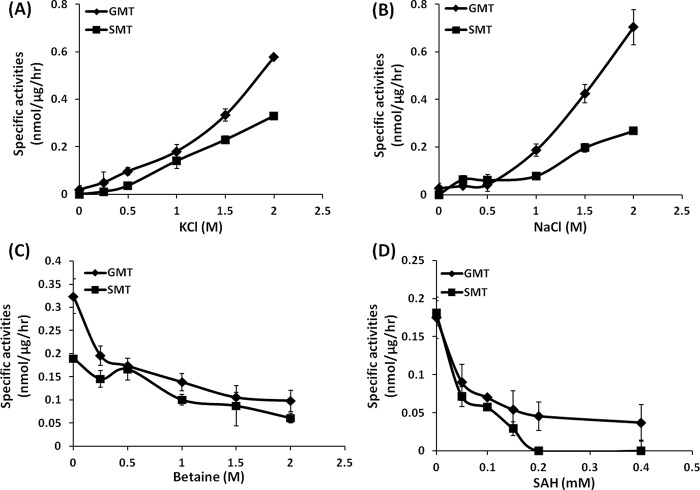
GSMT activities were activated by monovalent cations but inhibited by the end product betaine and the competitive inhibitor SAH. Assay mixtures contained 0.1 M TES, pH 7.3, 1 mM SAM (containing 0.1 μCi/2 pmol [3H-methyl]-SAM) and 0.5 M glycine or sarcosine under various concentrations of KCl (A) or NaCl (B). The inhibition assays were carried out under 1.0 M KCl with various concentrations of betaine (C) or SAH (D). All data points were averaged from three independent experiments.

In this study, native GSMT was purified to investigate this unique enzymatic trait and to verify that it was not caused by recombinant expression in *E*. *coli*. The results showed that both GMT and SMT activities were enhanced under high salt concentrations of KCl and NaCl (Fig 2A and 2B). Both the GMT and SMT activities of GSMT under the 2.0 M KCl condition were 30-fold higher than those without potassium ions (Fig 2A), whereas the GMT and SMT activities of rGSMT were enhanced 20- and 8-fold, respectively, under the 2.0 M KCl condition (S1 Fig). A previous study showed that rGSMT activities were also stimulated by a high concentration of sodium ions [11]; this is consistent with our results showing that both GMT and SMT activities from GSMT improved 25- and 4-fold, respectively, under 2.0 M NaCl compared with the condition without sodium ions (Fig 2B). This revealed that GSMT activities *in vitro* were regulated by monovalent cations, which have similar ion-protein interactions, even at the tested sodium ion concentration that was surfeit physiologically.

### Native GSMT was feedback-inhibited by betaine and competitively inhibited by SAH

Despite betaine being synthesized through step-wise methylation from glycine as an energy consumptive system, which requires three units of SAM to produce one unit of betaine, the amount of accumulated betaine still reached 0.76 M in *M*. *portucalensis* FDF1^T^ when it was grown under optimal conditions [25]. To conserve energy for *M*. *portucalensis* FDF1^T^ restoration after osmotic stress, the rate-limiting enzyme rGSMT was feedback-inhibited by the end product betaine and the competitive inhibitor SAH [11]. In this study, betaine inhibition assays were repeated with GSMT under the 1.0 M KCl condition, as described previously. The specific activities of GMT and SMT were inhibited to 43% and 53%, respectively, under the 1.0 M betaine condition but remained at 30% and 32%, respectively, with 2.0 M betaine (Fig 2C). This result indicated that when the intracellular amount of betaine reached 2.0 M, GSMT still retained more than 30% of its relative activity to produce betaine to survive in extreme hyperosmotic stress. Furthermore, the GMT activity of rGSMT, which performed without additional potassium chloride, was completely blocked under conditions with 1.5 M betaine to economize energy after stress adaptation [11]. This indicates that GSMT may decide to switch on or off the betaine synthesis process in accordance with the amount of intracellular potassium ions and the end product, betaine.

In the betaine biosynthesis pathway, SAH is a competitive inhibitor that is at a 3-fold higher level than end product betaine. Our previous study showed that the I_50_ values of SAH for GMT and SMT activities from rGSMT were 50 μM and 70 μM, respectively [11]. In this study, GSMT was also sensitive to SAH, whose I_50_ concentrations for GMT and SMT were 50 μM and 37 μM, respectively (Fig 2D). The sarcosine methylation activity of GSMT was 2-fold more sensitive to SAH than rGSMT. It is known that the betaine synthesizing gene cluster (BSC) contains SAM synthetase (*sams1*), SAH hydrolase (*sahh1*), *gsmt*, *sdmt*, and adenosine kinase (*adk*), with other copies of SAH hydrolase (*sahh2*) located downstream of the BSC (Lai and Lai, unpublished data). There are two copies of SAH hydrolases located up- and downstream of *gsmt* and *sdmt* in the *M*. *portucalensis* genome, which suggests that the amount of SAH produced by betaine biosynthesis will be immediately hydrolyzed to adenosine and homocysteine.

### Kinetic parameters of native GSMT compared with rGSMT

The kinetic parameters of rGSMT without additional potassium ions show significant lower kcat value to substrate glycine or sarcosine than the other homoenzymes [11] that represented ionic strength plays a crucial role to control the rate of chemical step. The lower catalytic efficiency (k_cat_/K_m_) of rGSMT in compare to rSDMT indicated GSMT was the rate-limited enzyme of this methyltransfer pathway. According to the ionic strength dependent activity of GSMT (Fig 2A and 2B), we hypothesized that ion-protein interaction is participated in the regulation of the protein-substrate interaction. In this study, the kinetic assays on rGSMT were repeated and compared with that of GSMT under the addition of 1.0 M KCl (Table 2). Both GSMT and rGSMT have similar catalytic efficiency (k_cat_/K_m_) that is quite lower than SDMT and rSDMT (Table 2).

**Table 2:** Kinetic parameters of betaine synthesizing enzymes from *M*. *portucalensis* FDF1^T^.

Enzyme^a^	Apparent K_m_ (mM)			V_max_ (nmol/min)	K_cat_/K_m_	Reference
	Gly.	Sar.	Dim.			
GSDMT	0.47			0.0020	0.010	Lai et al., 2006
		3.14		0.0060	0.005	
			2.45	0.0040	0.004	
GSMT^a^^b^	2190			0.0660	3.01 x 10^−5^	This study
		710		0.0232	3.27 x 10^−5^	
SDMT		2.29		0.8100	0.884	Chen et al., 2009
			3.76	4.8800	3.244	
rGSMT^b^	7500			0.3562	4.75 x 10^−5^	This study
		1410		0.0570	4.04 x 10^−5^	
rSDMT		0.66		0.4400	0.670	Lai and Lai, 2011
			0.66	0.7400	1.120	

a. The kinetic assays were performed in the condition with 1.0 M KCl.

b. The standard deviation was shown in Fig 3C.

### Potassium ions activated the reaction velocity of native GSMT and promoted the catalytic efficiency of rGSMT

Monovalent cations (K^+^ or Na^+^) act as activators to improve the activities of GSMT (Fig 2A and 2B) and rGSMT [11]. Our previous study showed that both K_m_ value of glycine and sarcosine of rGSMT were significantly decreased with high concentrations of potassium ions addition [11]. In this study, the detailed kinetic parameters of rGSMT under various concentrations of KCl in comparison with native GSMT were demonstrated (S2 Fig and Fig 3C). The K_cat_/K_m_[glycine] values of rGSMT increased from 1.14 to 6.88 following the shift from 0.5 M KCl to 1.5 M; therefore, the K_cat_/K_m_[sarcosine] values increased from 1.16 to 6.36 (Fig 3C). As predicted, GSMT has a similar tendency to that of rGSMT in catalytic efficiency under various concentrations of KCl. This phenomenon indicated that the rate of this chemical step was dependent on the ionic strength in reaction system. The kinetic analysis of GSMT (Fig 3A and 3B) and rGSMT (S2A and S2B Fig) both displayed classic competitive inhibition plots, demonstrating that both GSMT and rGSMT are competitively inhibited by SAH during the transmethylation reaction. Interestingly, the competitive inhibition from both GSMT and rGSMT was relieved in a dose-dependent manner (Fig 3A and 3B and S2A and S2B Fig). It is noteworthy that the potassium ions had different influences on native and recombinant GSMT and that GSMT displayed a higher turnover rate to release the product, whereas rGSMT possessed a higher catalytic efficiency to the substrate glycine. Based on the above kinetic analysis, we hypothesize that the post-translational regulation of GSMT from original *M*. *portucalensis* FDF1^T^ may differ from rGSMT, which was overexpressed in *E*. *coli*.

**Fig 3:**
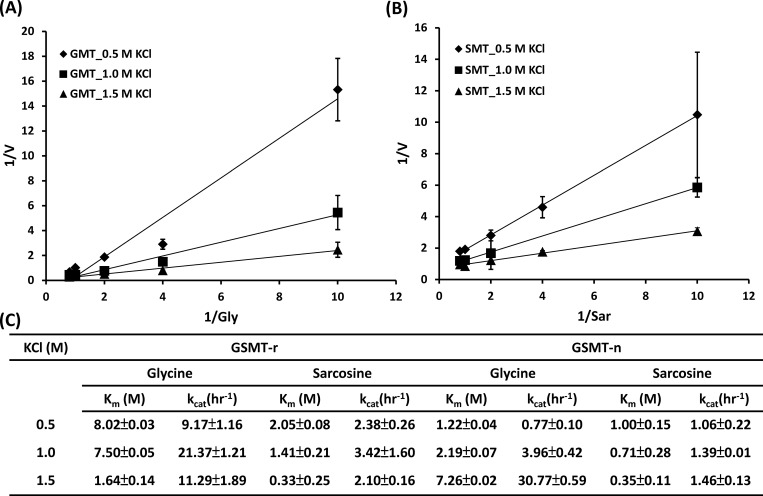
The reaction velocity of GSMT was significantly promoted under higher concentrations of KCl. Lineweaver-Burk plots of GSMT under various concentrations of KCl with glycine (A) or sarcosine (B) as the substrate. (C) Kinetic parameters of GSMT and rGSMT under 0.5 to 1.5 M KCl conditions. All data points were averaged from three independent experiments.

### Differential phosphorylation between native GSMT and rGSMT

Our preliminary phosphoproteomic analysis of *M*. *portucalensis* FDF1^T^ revealed 6 unambiguous phosphorylation sites on GSMT (S1 Table) [19]. Further, the phosphorylation-mediated modification on purified rGSMT was detected through the same phosphopeptide preparation process. A total of 15 reliable phosphosites was identified on rGSMT, with five conserved phosphosites detected on GSMT (S1 Table). The differential phosphorylation suggests that the substrate specificity of phosphatase from *M*. *portucalensis* FDF1^T^ may differ from that of *E*. *coli*. This phenomenon led us to investigate the phosphorylation levels of GSMT and rGSMT. We therefore extended our study to observe the level of phosphorylation of both GSMT and rGSMT through mass spectrometry. The results revealed 23.1% stoichiometry phosphorylation site occupancy in rGSMT (S2 Table); however, there was no detectable phosphorylated peptide from native GSMT. Therefore, the loss of a modified phospho-group may be due to the complicated purification process. Based on the above evidence, the variation in kinetic parameters between GSMT and rGSMT may be associated with phosphorylation-mediated post-translational modifications.

## Conclusion

In this study, we optimized the purification procedure of GSMT from *M*. *portucalensis* FDF1^T^. A total of 0.4 mg of GSMT was obtained from each batch of 4.8 L cultures. Both native GSMT and rGSMT have similar traits that are activated through monovalent cations, feedback inhibition by the end product betaine and competitive inhibition with SAH. Comparisons between native and recombinant GSMT revealed that GSMT has a higher sensitivity to SAH than rGSMT. Otherwise, the potassium ion-mediated modulation on the kinetic assays indicated that the turnover rate of the GSMT product was enhanced, whereas rGSMT possessed a higher efficiency to transfer the methyl group to the substrate glycine. The different phosphorylation sites and the variation in post-translational modifications of phosphorylation between GSMT and rGSMT may be due to differential phosphorylation-mediated modifications. This study provides a new angle to investigate regulation of the osmolyte synthesis process by modulating GSMT via post-translational phosphorylation.

## Supporting Information

S1 Table Identified phosphorylated sites on GSMT and rGSMT. (PDF)

S2 Table The relative quantitation of non-phosphopeptides from rGSMT treated with or without phosphatase. (PDF)

S1 Fig rGSMT activities were promoted by potassium ions. (PDF)

S2 Fig The kinetic assays of rGSMT. (PDF)
