# Supplementary material for: Comparison of Enzymatic Traits between Native and Recombinant Glycine Sarcosine N-Methyltransferase from Methanohalophilus portucalensis FDF1T
Source: PLoS One. 2016 Dec 30;11(12):e0168666. doi: 10.1371/journal.pone.0168666 (PMC5201303; doi:10.1371/journal.pone.0168666)
Supplement: S1 Table — (PDF) [file pone.0168666.s001.pdf]

S1 Table. Identified phosphorylated sites on GSMT and rGSMT.

Protein source	p-site	Score	Amino acid	Modified sequence	Phospho (DHSTY) sites Probabilities
GSMT	46	117.53	S	_AE**pS**EGDTIINILK_	AES(1)EGDTIINILK
	68	149.82	T	_VLDVA**pT**G**p[T]**GFNSVR_	VLDVAT(0.882)GT(0.116)GFNS(0.002)VR
	70	155.94	T	_VLDVATG**pT**GFNSVR_	VLDVATGT(1)GFNSVR
	169	115.91	Y	_N**pY**DAILDDGYSSK_	NY(0.98)D(0.02)AILDDGYSSK
	178	223.93	S	_NYDAILDDGY**pSp[S]**K_	NYDAILDDGY(0.001)S(0.803)S(0.196)K
	179	161.2	S	_NYDAILDDGYS**pS**K_	NYDAILDDGYS(0.041)S(0.959)K
rGSMT	46	127.91	S	AE**pS**EGDTIINILKER	AES(1)EGDTIINILKER
	50	181.32	T	AESEGD**pT**IINILKER	AESEGDT(1)IINILKER
	68	148.44	T	KVLDVA**pT**GTGFNSVR	KVLD(0.06)VAT(0.92)GT(0.019)GFNS(0.001)VR
	70	156.13	T	VLDVATG**pT**GFNSVR	VLDVAT(0.011)GT(0.989)GFNSVR
	74	114.78	S	KVLDVATGTGFN**pS**VR	KVLDVAT(0.056)GT(0.022)GFNS(0.922)VR
	106	62.338	Y	AFDNARDHG**pY**LMR	AFDNARDH(0.004)GY(0.996)LMR
	169	35.803	Y	N**pY**DAILDDGYSSK	NY(0.906)D(0.089)AILD(0.002)D(0.002)GYSSK
	174	147.7	D	NYDAIL**pD**DGYSSK	NYDAILD(0.964)D(0.027)GY(0.007)S(0.001)S(0.001)K
	179	132.9	S	NYDAILDDGYS**pS**K	NYDAILDDGYS(0.029)S(0.971)K
	188	81.113	D	NYDAILDDGYSSKHAHYYCG**pD**TVSVYPEHVDEGLAR	NYDAILDD(0.001)GY(0.003)S(0.001)S(0.001)KH(0.001)AH(0.012)Y(0.013)Y(0.049)CGD(0.879)T(0.019)VS(0.015)VY(0.005)PEHVDEGLAR
	191	109.13	S	HAHYYCGDTV**pS**VYPEHVDEGLAR	HAHYYCGDTVS(0.989)VY(0.01)PEHVDEGLAR
	193	86.179	Y	HAHYYCGDTVSV**pY**PEHVDEGLAR	HAHYY(0.001)CGD(0.025)T(0.022)VS(0.027)VY(0.922)PEH(0.001)VD(0.002)EGLAR
	212	81.48	S	FKYEFSDG**pS**VYNLNMFPLRK	FKYEFS(0.021)D(0.017)GS(0.958)VY(0.004)NLNMFPLRK
	214	67.902	Y	FKYEFSDGSV**pY**NLNMFPLRK	FKYEFSDGS(0.001)VY(0.999)NLNMFPLRK
	247	92.427	T	E**pT**YKEDEPDFFLHVAEKN	ET(0.822)Y(0.155)KED(0.024)EPDFFLHVAEKN

All of the phosphorylation sites were determined in each peptide with >99% identification confidence (p > 0.75). Phosphorylation sites with a localization

probability of at least 0.75 assigned by MaxQuant software are defined as unambiguous p-sites which indicated in bold and red for Ser, Thr, Tyr and blue for Asp.
