# Supplementary material for: Comparison of Enzymatic Traits between Native and Recombinant Glycine Sarcosine N-Methyltransferase from Methanohalophilus portucalensis FDF1T
Source: PLoS One. 2016 Dec 30;11(12):e0168666. doi: 10.1371/journal.pone.0168666 (PMC5201303; doi:10.1371/journal.pone.0168666)
Supplement: S2 Table — (PDF) [file pone.0168666.s002.pdf]

S2 Table. The relative quantitation of non-phosphopeptides from rGSMT treated with or without phosphatase.

phosphatase	Peptide sequence	m/z	Charge	Retention time	Peak area	Phospho-peptides[a]
Treated	KVLDVATGTGFNSVR	782.4281	2	35.46	15,729,371,424	23.1%
Untreated	KVLDVATGTGFNSVR	782.4281	2	35.23	12,093,191,862	

a. The differences of peak areas between treated- and untreated phosphatase from KVLDVATGTGFNSVR indicated the peak area of phosphorylated peptide. The result of phosphorylation stoichiometry was calculated by peak area of phosphorylated “KVLDVATGTGFNSVR” divided by peak area of condition treated with phosphatase.
