# Supplementary material for: Comparison of Enzymatic Traits between Native and Recombinant Glycine Sarcosine N-Methyltransferase from Methanohalophilus portucalensis FDF1T
Source: PLoS One. 2016 Dec 30;11(12):e0168666. doi: 10.1371/journal.pone.0168666 (PMC5201303; doi:10.1371/journal.pone.0168666)
Supplement: S1 Fig — (PDF) [file pone.0168666.s003.pdf]

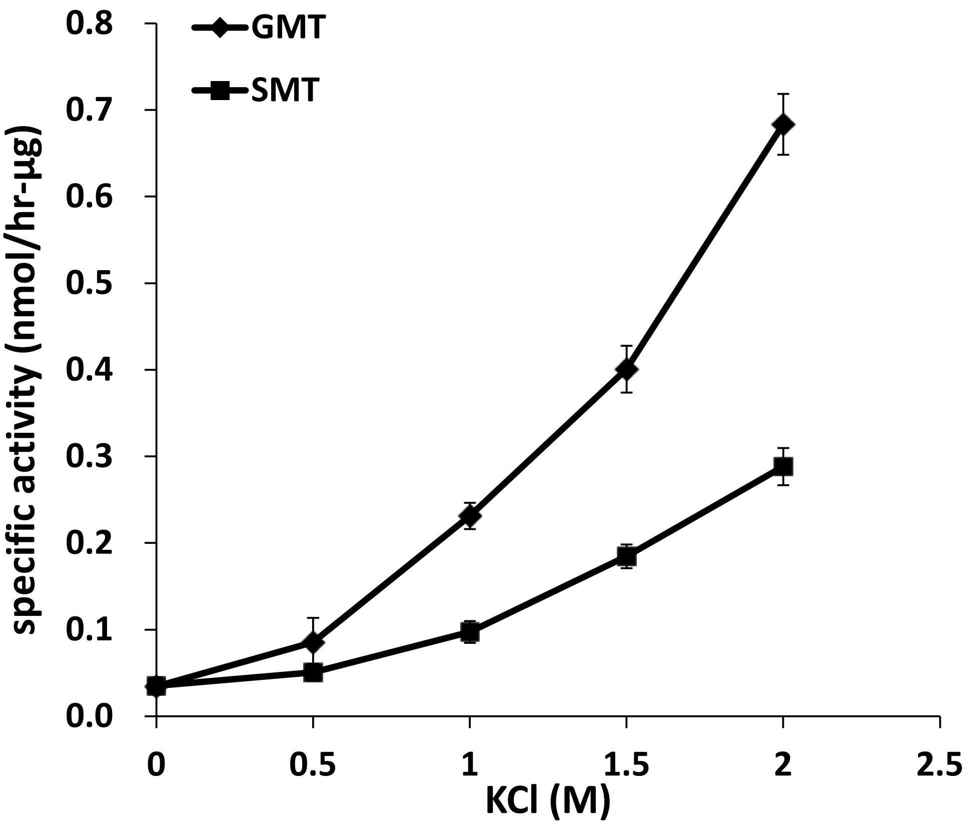

S1 Fig. rGSMT activities were promoted by potassium ions. Assay mixtures contained 0.1 M TES, pH 7.3, 1 mM SAM (containing 0.1 μCi/2 pmol [^{3}H-methyl]-SAM) and 0.5 M glycine or sarcosine under various concentrations of KCl. All the data points were averaged from independent triplicate experiments.
