# Supplementary material for: Comparison of Enzymatic Traits between Native and Recombinant Glycine Sarcosine N-Methyltransferase from Methanohalophilus portucalensis FDF1T
Source: PLoS One. 2016 Dec 30;11(12):e0168666. doi: 10.1371/journal.pone.0168666 (PMC5201303; doi:10.1371/journal.pone.0168666)
Supplement: S2 Fig — (PDF) [file pone.0168666.s004.pdf]

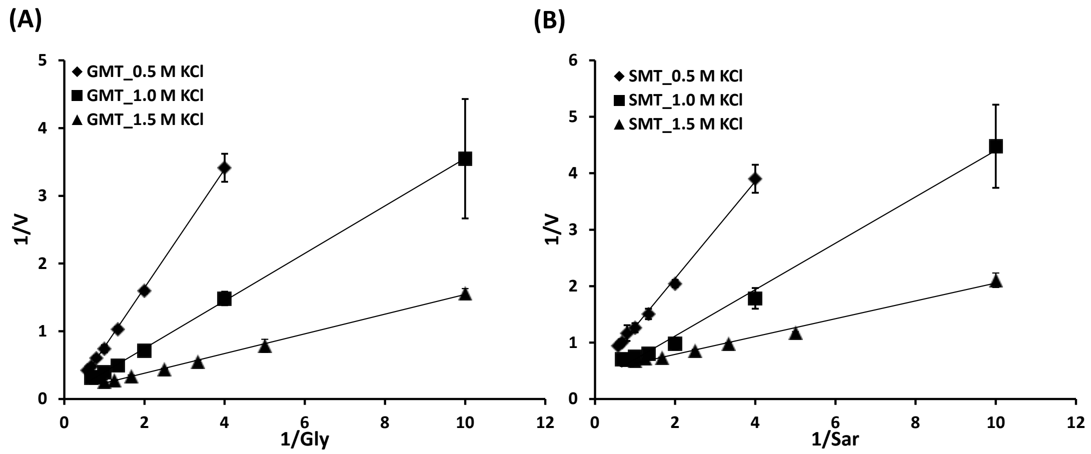

S2 Fig. The kinetic assays of rGSMT. Lineweaver-Burk plots of rGSMT under various concentrations of KCl with glycine (A) or sarcosine (B) as substrate. All the data points were averaged from independent triplicate experiments.
